# "The Hospital" Nursing Mirror

**Published:** 1901-08-31

**Authors:** 


"The Hospital" Nursing Mirror.

Being the Nursing Section of "The Hospital."

[Contributions for this Section of "The Hospital" should be addressed to the Editor, "The Hospital" Nursing Mirror, 28 & 29 Southampton Street Strand, London, W.C.]

### Notes on News from the Nursing World.

### THE DUKE AND DUCHESS OF CORNWALL AT CAPETOWN.

One of the most popular acts of the Duke and Duchess of Cornwall at Capetown was the performance on Thursday, last week, of the ceremony of laying the foundation stone of the nurses' home which is being erected to the memory of Queen Victoria. The function which took place at noon only occupied a few minutes, and was of the simplest character. But a large gathering assembled and gave the Duke and Duchess an enthusiastic greeting. It is well known that the Heir Apparent and his wife take the greatest interest in all matters affecting the welfare of nurses, either in Great Britain or in our possessions beyond the seas, and there is no doubt that the little ceremony at Capetown afforded them as much pleasure as some of the more imposing incidents of their tour.

### THE DEMAND FOR ENGLISH NURSES BEYOND THE SEAS.

As our columns this week testify, it is not only in British Columbia that there is an opening for English nurses. A contributor who has had experience in Buenos Aires, and whose account of her first case in an Argentine family will be read with amusement, shows that there is scope in that quarter of the world for trained nurses of courage and capacity—at any rate if they are willing that their work should consist principally of maternity cases; while a correspondent whose lot it has been to nurse in another part of South America refers to the need of nurses, "especially of midwives." At some of the Brazilian ports, of course, there are drawbacks, but they will not seem insuperable to those who possess capital as well as brains and enterprise. The openings for maternity nurses in other countries may turn the attention of many to this branch of the profession, though the account which a nurse who entered a maternity hospital for training sends us indicates that the period of probation is not always pleasant. It should, however, be remembered that the provision for the nurses in well-ordered lying-in institutions is equal in comfort and other respects to that of the most important general hospitals.

### RECOVERY OF SISTER WEBSTER.

It is announced that Nursing Sister G. H. Webster was discharged from hospital to duty in the week ending August 18. Miss Webster has been ill for some weeks in South Africa, and her condition at one time caused serious anxiety to her friends. We congratulate them and herself upon her complete recovery.

### RETURNED NURSING SISTERS.

The following nursing sisters arrived in the *Assaye*, which reached Southampton from South Africa on Monday:—Nurses H. A. Lawrence, M. B. Pertwee, M. Roche, E. F. Upperby, B. L. Shappere, and B. E. Barrow. The nurses in charge of the invalids were Nurses H. Hogarth and E. M. Sultan. The *Wakool*, which left Cape Town on August 21 for England, has on board Sisters E. Mackae, A. S. Clarridge, K. M. Picot, and K. Forde.

### PRINCE ALFRED HOSPITAL, SYDNEY.

The nursing equipment of the Queen Victoria Memorial Pavilions which are intended to flank the main entrance on the right and left of the Prince Alfred Hospital, Sydney, and will provide accommodation for a hundred additional patients, will cost £15,000. A correspondent informs us that £10,000 of this sum has already been raised, and that the remainder will be forthcoming before the money is actually needed. "The pavilions," she continues, "will harmonise in design with the rest of the hospital, with the graceful arches and wide balconies which protect the wards from the fierce sunshine of summer and provide a spacious promenade or lounging place for the convalescents." Miss McGahey, the matron of the hospital, is now in England, and, we have no doubt, will be glad to furnish any information which may be desired respecting the nursing.

### "TIGHT-LACED, CURLY-HEADED NURSES."

In a "Diary of a Nurse in South Africa," Madame Bron complains that when she was engaged in nursing the Boers, "tight-laced, curly-headed nurses, who preferred flirtation to hard work," got in her way. The inference is that "the plague of women" was not confined to one side. There is no reason why a nurse should not possess curly hair, and even tight-lacing is not a crime, although it detracts seriously from a nurse's powers of endurance. But if any of the nurses sent out by the Dutch and Belgian Red Cross Association showed a preference for flirtation rather than for hard work, they were obviously unfitted for their duties; and we are not surprised that Madame Bron was glad to be formally dissociated from them.

### WORKHOUSE NURSING IN IRELAND.

The new order issued by the Irish Local Government Board, which we cited in full last week, has naturally given rise to a great deal of controversy in the sister island, and this week an important conference is being held at Dublin of representatives of the various Poor Law Boards in the country, and of the governing bodies of the infirmaries. We shall publish a special report of the proceedings next week. Meanwhile, the action of the Irish Local Government Board, and the assurances of Mr. Wyndham in the House of Commons before the prorogation, that the circular is not to be regarded as the final answer to the demands for improvements in the system of nursing in workhouse infirmaries on the other side of St. George's Channel, have been received with satisfaction in many quarters, and with anger in some. Of course the ratepayers are being warned that the reform cannot be effected without imposing an additional burden upon them.

### RESIGNATIONS AT CROYDON INFIRMARY.

We understand that six nurses have resigned their position at Croydon Workhouse Infirmary since the appointment of a superintendent, and it appears that more are imminent. From the first the manner in which Miss Julian, the esteemed matron, has been treated by the Board of Guardians has been much resented by the members of the nursing staff, and since the election of a new official this feeling has increased rather than diminished. It was not expected that, in the circumstances, the applicants for the position of superintendent of nurses would be numerous, and the guardians probably made the best choice they could. They must, however, be chiefly blamed for the friction that still obtains in the infirmary, though the lady who accepted service under them must have known, when she undertook her existing obligations, the opinion of the nursing world in reference to the proposed re-arrangement of duties.

### BOTTLE-FED BABIES.

In another column a "Mother" enlarges on the great need for maternity nurses to add to their training a knowledge of at least the most approved methods of bringing up a delicate baby by hand. It seems likely that a wider experience on this subject will in future be required, not only in England but also on the Continent. For some years British women have manifested an increasing desire to get rid of the trouble of feeding their babies, and have resorted to bottles, whilst French and Italian mothers have had recourse to the assistance of a wet nurse. The French authorities, believing that this custom is not only injurious to the foster-mother's own offspring, but to her foster-children, and also a cause of the depopulation of the country, have issued through the Prefect of Police in Paris a decree that no mother shall be allowed to take a situation as a wet nurse until her own child is seven months old. This will have the effect of augmenting the number of bottle-fed babies, and English maternity nurses, who think of settling in France, will do well to remember that it will probably fall to their lot to inaugurate a new system of feeding among French children, about which, unless they have been in practice some years, they are themselves almost in ignorance.

### SUNDERLAND DISTRICT NURSING ASSOCIATION.

We are glad to learn that the workpeople in the shipyards and other industries on the Wear are taking a great interest in the movement for promoting the endowment of the Sunderland District Nursing Association. Towards this fund between £3,000 and £4,000 has already been raised, and though chiefly in large amounts, such as a contribution of £100 from the Ecclesiastical Commissioners, a considerable measure of support has been given by the artisan class in the shape of weekly subscriptions of small sums. This shows that the latter thoroughly appreciate the labours of the nurses of the Association among them.

### THE NEW NURSES' HOME AT SCARBOROUGH.

The nurses of Scarborough Hospital are to be warmly congratulated upon the admirable provision made for their comfort in the new home, which has been opened by the Archbishop of York. The building has been erected at a cost exceeding £2,000, of which the whole has been raised, thanks largely to a grant of £1,000 from the Marriott Trust Fund and to a subscription of £500 from the Misses Hugill in memory of their father. The home, which has direct communication with the hospital, consists of a large sitting or recreation room on the ground floor, a small service pantry, twelve bedrooms, three bath rooms, lavatories, etc. It is built of red brick, and has a very substantial looking appearance. It is well lighted, and is supplied with hot water. In his speech at the opening function the Archbishop compared the nurses of to-day with those pictured in the pages of Charles Dickens and other writers, and said that the improvement had been wonderful—"The whole work had been lifted on to a higher level than it was half a century ago."

### DEATH OF A NURSE IN A TRAIN.

The death of Miss Annie Cotterell, a nurse of 34, under very distressing circumstances, is reported. Miss Cotterell had left her home at Preston on Saturday, the 17th, in order to proceed on the 19th to South Africa for duty. She travelled from Preston to Stafford with her sister, and seemed somewhat depressed at the prospect of leaving her friends. Soon after the train left Stafford she suddenly fell forward, and upon the arrival of the train at Rugby it was found that life was extinct. An inquest was held subsequently, and the medical evidence was to the effect that death was due to failure of the heart, accelerated by the excitement. In the course of the proceedings it transpired that her heart was known not to be strong, but that in spite of this fact she had been certified as fit for work in South Africa. It was not stated by whom, or at what date, Miss Cotterell was examined, but no nurse suffering from any affection of the heart should be exposed to the excitement inseparable from service in time of war.

### MEALS UPON THE STAIR.

Two private nurses were recently engaged at a large public school nursing a boy who had had his leg amputated. As soon as he got a little better the wife of the master informed the nurses that she could no longer give them their meals in her husband's study, but they could have them on the landing. The doctor on being made acquainted with this proposed arrangement could scarcely credit it, but finding on inquiry that it was really the case at once put a stop to it. The boy composed the following lines:—

I.

The topic of this ode I've laid
To tell the manner how
The nurses here were almost made
To dine like any cow!

II.

As soon as the patient's pulse was good
This problem did arise—
Where should the nurses have their food?
There were no barns nor sties!

III.

So Mrs. B. did ponder much—
Considered it quite fair
That nurses, being such-and-such,
Should feed upon the stair.

IV.

So two small troughs were soon brought up
And placed upon the floor:
From these the nurses were to sup
Outside the patient's door!

V.

When on this topic she did speak,
In accents low, they said—
"Well! Mrs. B., you *have* got cheek!
We'll chuck the case instead."

But they didn't.

### SHORT ITEMS.

At Torquay regatta on Monday a brake containing convalescent soldiers staying at Syracuse Home met with an accident. The matron of the home, who was with the patients, had her thigh and arm broken.

### Lectures to Nurses on Medicine and Medical Nursing.

By NORMAN DALTON, M.D. London, F.R.C.P., Physician to King's College Hospital.

### LECTURE XVIII.—DISEASES OF THE KIDNEY.

### THE URINE.

To the naked eye the kidney appears to be a solid organ, but it is really made up of a large number of minute tubes which twist about and are closely packed one against the other. At its far end, each tubule is expanded so as to form a round capsule in which lies a round mass of twisted blood vessels, which is called the "Malpighian" body or tuft. At its other end each tubule opens into the pelvis of the kidney, a triangular hollow space occupying the hilum of the organ. The solid matter, such as the urea, which is found dissolved in the urine, is excreted by the cells which line the tubules while the tufts excrete water. So the principle is like that of a water closet, *i.e.*, the water is poured out at the top of the tubule, and as it runs down the tubule, it washes away the urea produced in the tubule and carries it to the pelvis of the kidney, whence it passes through the ureter into the bladder. The urea is easily dissolved so that the urine is quite clear. Inflammation of the kidney is called "nephritis," and inflammation of the pelvis of the kidney is called "pyelitis." These terms will be used for the future.

The "urine" is a clear yellow fluid. It is acid, and when the urinometer is put into it a specific gravity of about 1015 should be registered. The normal quantity passed is about 50 oz. per day. This quantity is diminished if very little fluid be taken in the 24 hours, and also in hot weather and during an attack of diarrhœa, because much water is taken off in the sweat and in the motions respectively. It is also diminished when "shock" or "collapse" is present, as occurs in strangulation of the small intestine; and, after operations on the urethra or bladder, the quantity may be reduced to a minimum. When "back pressure" sets in, in cases of mitral disease, the urine becomes scanty, and the same thing happens in every case of "fever." In "acute nephritis," especially when due to scarlet fever, the quantity passed may be reduced to an ounce or two a day, or the secretion may be entirely suppressed. It is important to distinguish between suppression of urine and retention. In the former case no urine is made, while in the latter the urine is secreted but cannot be passed on account of a stricture of the urethra, or of paralysis of the bladder, etc. Complete suppression of urine is most often met with in acute nephritis or after an operation on some part of the urinary tract. But it is occasionally caused by stone in the kidney. It is usually found in such cases that one kidney has been disabled for years by the impaction of a calculus in the ureter, and that the other kidney has become suddenly affected in the same way, so that no urine can reach the bladder.

When the quantity of urine passed per diem is larger than normal, the condition is called "polyuria." This occurs when an excessive quantity of fluid is drunk, especially such fluids as coffee, tea, and alcohol. It also occurs in cold weather and in consequence of depressing emotions, such as fear. But it is a constant symptom of certain serious diseases, for example the form of Bright's disease called the granular kidney, and diabetes. There are two forms of diabetes. In one, which is called diabetes mellitus, the urine contains grape sugar, while in the other, diabetes insipidus, no sugar is present. The former is, of course, by far the more serious affection. Many anæmic and hysterical girls pass a great deal of urine, and may be said to have a mild form of diabetes insipidus.

Nurses must remember that when it is necessary for the physician to estimate the quantity of urea, or of sugar in the urine, it is incumbent on them to collect and mix together every specimen of urine which is passed during the 24 hours. The reason is that each specimen does not contain the same amount of urea or sugar; so that if only one specimen were tested, the quantity it yielded might be greater or less than the others which would make the estimate for the 24 hours wrong. The whole amount of urine passed in the 24 hours can only be collected by explaining to the patient what is wanted and obtaining his co-operation, and even then some is usually lost with the fæces. In reporting the total quantity of urine passed per day, nurses must also report the number of motions, and if these have been relaxed; and, also, if profuse sweating has occurred. It is in cases of diabetes and of Bright's disease that the quantitative examination of the urine most often becomes necessary. But it is also necessary after operations on one kidney, especially when the whole of one kidney has been removed for tumour or tuberculosis, for it is essential to know if the other kidney is doing the work of both. It is very remarkable how soon one kidney becomes capable of doing double work, and, as a matter of fact, in these cases, the remaining kidney grows to nearly twice its former size, new and perfectly formed kidney tissue being developed in it.

The "colour" of the urine tallies with the quantity secreted, so that in fever, mitral disease, acute nephritis, etc., the colour is dark; while in "polyuria" from all causes it is pale. Care must be taken to distinguish the deepening of the natural colour of the urine such as is seen in fever and mitral disease, from the dark colour which occurs when other colouring matters, such as bile or blood, are mixed with the urine. If there be much bile present it will be easily recognised by any nurse who has seen the same condition before, more particularly as it stains linen such a definite yellow. Slight traces of bile, on the other hand, can only be recognised by chemical tests, so that all that a nurse can do is to draw the attention of the physician to any specimen which she may suspect of containing bile. Blood, also, if it be present in very small quantity, can only be recognised by chemical tests, or by the microscope, or the spectroscope. If the blood be coming from the kidney substance itself, and be in considerable quantity, the urine has a dark appearance, like smoked glass, for which reason it is called "smoky" urine, but if the quantity be very large, it becomes quite as dark as "porter." In many cases the blood is easily recognised as blood, particularly if it be coming from the bladder. Some drugs change the colour of the urine; for instance, santonin, when given by the mouth, may turn the urine yellow or orange.

The specific gravity of the urine is usually inversely proportional to the quantity passed, so that it is high in fever, acute Bright's disease, etc., and low in polyuria. But there is one great exception to this rule, namely, in diabetes mellitus, the quantity is abundant, and the specific gravity very high. Nurses are often called upon to test the "reaction" of the urine. In health it is acid, and in fever, gout, rheumatism, etc., it may be extremely acid, turning the blue litmus paper a bright red. After being passed, the urine tends to become alkaline because decomposition takes place in it. Therefore, the litmus paper should be applied as soon as it is passed. Occasionally it is found that the urine is alkaline, even when it leaves the bladder. This is most often seen in patients who are greatly debilitated, and such urine usually contains phosphates. In cases of small calculi in the pelvis of the kidney, physicians often prescribe alkaline salts, such as the acetate or citrate of potash which make the urine alkaline, or at any rate less acid, the idea being that the alkaline urine may dissolve the stone. If the drug made the urine too alkaline the result would be injurious, and consequently a nurse may be called upon to test every specimen passed with litmus paper, and to record whether it is faintly acid or alkaline. When pus becomes mixed with the urine in the pelvis of the kidney or in the bladder, the urine may become alkaline before it is passed, and is frequently very offensive.

### Beyond the Seas.

### NURSING AT BUENOS AIRES.

By an English Nurse.

There is no doubt that nursing in Buenos Aires is in a chrysalis stage. With a population of 800,000, and about 30,000 of them English, this place would appear at first sight a happy one for nurses. But she must be a dauntless, courageous, tactful woman, who would nurse under the trying conditions of a country where the nursing has been hitherto done by women of the servant order. By the "servant order" I mean women who, though gifted with a certain gentleness of manner are shockingly ignorant, some of them not even able to tell the time, and having great difficulty in reading and writing. No wonder patients are a little puzzled when they have to deal with an educated woman. With Argentines lady nurses are a complete novelty.

### My First Case in an Argentine Family.

The patient had been confined two days before my arrival, but her first nurse, she informed me, had been obliged to leave her suddenly. After the preliminary conversation was over the patient's husband asked me what I wanted for the night? I answered, "Some milk, etc., for the patient and a bed for myself." He replied, "How will you hear the baby crying if you are asleep?" He seemed astounded that I required any sleep at all. "I think I shall manage to wake," I answered, feeling inwardly amused, and thinking I could guess the reason of the sudden departure of the previous nurse, who, being of the servant type, was too diffident to mention that she needed sleep. I was determined to stay whatever happened, and pave the way for the next nurse who crossed the path of this particular family. The following morning the housemaid appeared and told me that coffee was ready in the kitchen, so I had to explain that my meals must be brought to me on a tray, then I had to say that I must be called Miss, or Nurse, or the servants would call me by my Christian name. After a couple of weeks we all got on splendidly. The patient's relations admired my uniform very much, especially the cap. When the mother came she brought two small children, aged six and seven, and passed them on for me to take care of for the first few visits, but I called the housemaid and told her to keep an eye on them, and they were never put under my charge again.

### The Asistencia Publica.

There are no Argentine training schools. The British Hospital gives its nurses a training course and lectures. The Government hospitals generally have a sister of charity as head of the wards, and two or three servants under her who do the nursing. The servants are not bound to stay any specified length of time. If they remain a long time they learn more about the care of the patients; if a short time they do not learn as much, and it is all the more uncomfortable for the patients. Some of the women are more enterprising. They take a course at the "Asistencia Publica," or "Public Assistance," a large government dispensary. Only out-patients are treated, and if they require any other care, they are sent to one of the numerous hospitals, of which there are many handsome in appearance, well equipped, but very badly managed. The dispensary course is two months at the dispensary, and four months spent in different hospitals, medical, maternity, etc., making a six months' course altogether. There are lectures twice a week, which the candidates for certificates are obliged to attend, consisting of "first aid to the injured," and making charts, etc. Anyone with a common school education, and average brains, might pick up a great deal in this course, but the usual run of women who take it are sadly lacking in these qualities. I greatly admired the patience of Dr. Cecilia Grierson, the first woman who has studied medicine in this country, and who gave the lectures I attended. She seemed in a quite wonderful manner to keep in touch with the not very promising audience. The six months' course completed, the competitors pass a simple exam., and are full-fledged nurses.

### The Question of Fees.

Private nursing pays well here if nurses are willing that their work shall consist principally of maternity cases, and provided they speak some Spanish, the language of the country. Even with English patients the servants are generally Spanish. For maternity cases the usual fee is 200 dollars a month (a sovereign is about 12 dollars), 5 dollars a day for extra days, if the nurse remains longer than the month. For other cases 7 dollars a day. The patient pays the nurse's washing and travelling expenses. There are about a dozen English trained nurses here. There have been a great many from time to time but they often go away disgusted, because they say they forget all they know only taking maternity cases. In private nursing about 80 per cent. are of this class. It is no good depending on doctors for work; now and then a doctor sends for a nurse, but as a rule the patients engage the nurse themselves, and one patient recommends a nurse to another. This seemed queer to me when I first arrived from New York where I had been private nursing for three years, and getting cases principally from doctors.

### The Cost of Living.

The expenses of living here are much greater than in England, just about double; clothing is very expensive, also house rents. A friend of mine and I share a room together in the heart of the city; horse cars, and electric trams pass the door. We pay 50 dollars a month between us, supplying all our own furniture. The room is 18 feet long and 12 wide, with a nice balcony, and a fire-place. Living economically costs with board from 80 to 90 dollars a month. It takes some time to become known, and I would not advise any nurse to come here unless she has capital to live independently of nursing for at least a year. Institutions pay nurses from 25 to 100 dollars a month, according to the position they occupy. The patient's relations are the greatest difficulty in nursing here. Families are very united, and insist on remaining in the room, no matter how much one may try and impress upon them that they are exciting the patient. They only speak of the patient as "poor little one," and do not dream of moving. This is supposed to be a temperate climate, but in winter the cold is sometimes intense, and in summer the heat is very great. It never snows, but the wind is bleak and cutting, which is trying to new comers, especially as the houses are built round a tiled "patio," or yard, the bedrooms communicating, but to reach the kitchen it is almost always necessary to go outside.

### To Nurses.

We invite contributions from any of our readers, and shall be glad to pay for "Notes on News from the Nursing World," or for articles describing nursing experiences, or dealing with any nursing question from an original point of view. The minimum payment for contributions is 5s., but we welcome interesting contributions of a column, or a page, in length. It may be added that notices of entertainments, presentations, and deaths are not paid for, but, of course, we are always glad to receive them. All rejected manuscripts are returned in due course, and all payments for manuscripts used are made as early as possible after the beginning of each quarter.

### Nursing at Warmbad, Transvaal.

By a Member of the Army Nursing Service Reserve.

Just two months ago to-day (July 24) another nursing sister and myself left Pretoria for "Warmbad," now more generally called "Warmbaths." The train departing early in the morning we entrained the night before. Pretoria Station is not a very enviable place to sleep in. As the train travelled north we noticed a great difference between the Bush Veldt and the High Veldt. Some of the mimosa trees are about eight or ten feet high, the average not more than three or four. Another variety, having long white thorns from one to three inches in length, grows very thickly, and is used for fencing-in cattle kraals. It keeps cattle in as well as barbed wire, and certainly it is barbed enough. The kopjes do not stand out so distinctly as in other parts of the Transvaal, or Orange River Colony. They are more like continual ranges of mountains without any mountain peaks.

### The Waters.

On arrival we found that the place consists of a hotel and 145 bath houses only. This has been quite a famous winter resort. A great number of people camped in the vicinity and took the baths, which are supplied with warm water from a hot spring enclosed in a rotunda-shaped building, where there are steam baths, all well fitted up with porcelain tubs, etc. Mr. Kruger spent on behalf of the Government £23,000 in improvements. The baths are supposed to be especially good for rheumatism. The following is an analysis of the waters by Dr. Hahn from one gallon of water:—

| | Grains. |
|---|---|
| Carbonate of soda ... ... ... ... | 16·84 |
| Chlo. of sodium ... ... ... ... | 9·168 |
| Carbonate of lithia ... ... ... ... | 0·12 |
| ,, lime ... ... ... ... | 1·82 |
| ,, iron ... ... ... ... | 0·29 |
| Sulph. magnesia ... ... ... ... | 0·36 |
| Silica ... ... ... ... ... ... | 2·45 |

Temperature varies from 120° to 140° Fahr.

### The Hospital.

The dining-room of the hotel which has been converted into a hospital, being large, makes a fine ward of 26 beds; the ladies' drawing-room, reading-room, and private dining-room are used for convalescents; and there are in all 80 beds. The billiard-room, with two fine billiard tables, has not been disturbed; the bar is our dispensary. The bedrooms and some suites are all in the compound, each opening on to a long "stoep" on either side of an arbour covered with vines. On the whole the hotel makes a very good hospital, having a large kitchen with two ranges. Major Hassard, R.A.M.C., has charge of affairs, and is without an assistant, but manages to get through a lot of work without any apparent difficulty. About once a month we have a hospital train up, which relieves us greatly, and by degrees we fill up again till the train returns. We keep all the bad cases however.

### Uniform always Worn.

The surrounding country has been well settled. There are very large farms which we have visited; they are all deserted now, but the houses have not been burned. They were evidently well furnished, nearly all furniture has, however, been destroyed, even the American organs being cut open. We often drive out in a donkey-cart to get lemons for the patients' lemonade. The Commandant objects to the men going, for the Boers frequently visit these places, and they are liable to be "sniped" at. If they have seen us, we have not had the pleasure of meeting them; we know they would do us no harm, therefore we are not afraid. We alwav wear our uniform, so they may know who we are.

### A Kaffir Kraal.

About five miles from here is a very large Kaffir Kraal called "Matalastadt." There are between five and six hundred inhabitants, and a good church. The houses are very neat, and are mostly surrounded with a wall made of mud which is decorated on the inside with grotesque designs of coloured clay. There is a very small entrance, and no windows. When the women and children come out to greet us they usually wear blankets, and are adorned with necklaces of beads, bracelets, and anklets. The piccaninnies are mostly nude. We have quite a number come in to be treated every morning; they love to take medicine, and usually bring in eggs as payment which we use for patients in hospital. One poor old woman came in 20 miles this morning to be treated.

### The Patients.

This is our second occupation of Warmbad; last August we only held it 18 days. We had not enough troops here so we retreated. We have had it garrisoned since March 31st, but it remains to be seen how much longer it will be necessary. At present we have frequent skirmishes all along the line up to Pietersburg. A train of provisions was taken about 10 days ago, 19 were killed and 12 wounded. A couple of days ago we had six wounded brought in and one Boer, a young fellow badly shot. One of our soldiers died next day. We have had several alarms; a surrendered Boer whose farm is a few miles from here came in a night or two ago for protection. He had been three hours under the bed in hiding while his wife cooked supper for some Boers visiting his farm. They took his horses and he came in on a donkey for which they had no use or it would have gone too without doubt. A commando of 600 had a laager near here at that time. It is rather amusing when there is an alarm. Two or three men who have been in commando but are now in employ of the British Government—hurry off to the camp to sleep for they know it will go hard with them if the Boers catch them on hospital ground. They are very bitter against those who have changed sides.

### Fears about Christmas.

The question still remains unanswered: How much longer will the war last? From month to month we have hoped that some understanding would be arrived at, and the Boers would surrender; but now it seems as far off as ever and I am very much afraid that we shall see another Christmas in South Africa.

### Presentations.

Camberwell Infirmary.—On Monday, August 19th, Miss M. H. Grace Russell, M.D., was presented with a silver salver by the medical and nursing staff of the infirmary. Dr. Russell's work won the regard of all in the infirmary, and her departure for New Zealand after a year's residence is regretted by everyone.

North London Consumption Hospital, Mount Vernon, Hampstead.—Miss May Foote, who for private reasons has just resigned her appointment at North London Consumption Hospital, Hampstead, has been presented with a pair of handsome prayer and hymn books in case from the patients, bearing the following inscription: "To Nurse Foote, from grateful patients of the Mount Vernon Hospital, August 15th 1901, as a token of their gratitude and esteem."

### Sick Nurses for Shipboard.

By a Voyaging Nurse.

It has often occurred to me during my many sea voyages in various parts of the world that the time has come when every big steamer carrying passengers should add a trained nurse to its permanent crew. Nowadays we regard skilled nursing as a necessity; it is no longer considered to be a luxury. The law has long required that every steamer accommodating passengers should carry a qualified doctor. And I hope before many years that the requirements will be extended so as to include a "sailor nurse."

### The Law as to French Vessels.

So long ago as 1887 a law was passed in France that all French passenger steamers should be equipped with proper hospital accommodation and that a trained nurse should be provided on each such vessel for the care of possible invalids. Now it must be confessed that the law so passed has not been strictly kept. I have been on passenger boats sailing under the French flag in the East and the "trained nurse on board" has been conspicuous by her absence. But it is a great advance for French legislators to have recognised the principle that skilled nurses are needed on shipboard.

### Sick Civilians at Sea.

A good deal of discussion has arisen of late as to the transport and nursing of sick soldiers at sea. I proceed to recite my own experiences and observations of the miseries that sick civilians suffer on board the big nurseless liners. It fell to my fortune some time since to go a-globe-trotting to Ceylon and India, and I shipped on board one of the splendid big British steamers plying between the Thames and the East. Since I am regarding the question of steamer transport from the invalid point of view, I will pass over the outward voyage, when we were a more or less hearty, healthy, sea-going crew, capable of putting up with unnecessary restrictions, not, perhaps, without grumbling, but at least without any harm being done. It is with the return voyage, when many of us were unable to show a clean bill of health, and several Indian invalids were aboard, sent home, some of them, as a last resource from finding a tomb in the tropics, that I have to deal. How they fared is the plain tale I propose to tell, without adding dramatic detail or colouring. I happen to know wherein the hardships lay, for I was myself a bad case of dysentery "ordered home" for the benefit of the voyage and because a further stay in the tropics was a matter of pressing danger. I will group myself among the three worst cases, one other being a relapsing typhoid, complicated with malaria and a tendency to lung trouble, the other a lady who had recently suffered a serious miscarriage. We left the tropics just as the rainy season set in—the weather was of that stifling, sultry kind which those familiar with the East know invariably accompanies the onset of the rainy season. Naturally, none of us was well enough to "dress for dinner," nor in a fit state to sit out a long meal in the sultry saloon, neither was the dietary fitted to our weakened digestions.

### No Punkahs.

At the sound of the dinner gong on the first night out, therefore, each of us remained reclining at full length in the deck drawing-room, where the movement of the ship ensured a certain languid breeze. Remember, we had just come from a land abounding in punkahs. For some unexplained reason no punkahs are provided on this "crack line." We ran the gauntlet of the tropics and the Red Sea with never a punkah to refresh our weary, flagging bodies. Ringing up the deck steward, each asked for mutton broth, rice pudding, or some form of invalid dinner. "No food or drink can be served out of the saloon without special orders from the doctor" was the ready response. The doctor was dining. We hesitated to disturb him. Regarding it as an emergency case, we apologetically asked the doctor to leave his meal. He came promptly and pleasantly, and ordered a *menu* suited to our sickly and miserable condition. I want to emphasise the fact that none of us were *mal de mer* victims. Each was suffering from a grave constitutional illness. Relieved by the dictum of the doctor we anticipated pleasurably our little meal, feeling somewhat exhausted by the small formalities which had delayed our dinner to an unusual lateness.

### The Rules of the Company.

"Oh," said our friend the deck steward, "will you take your soup in your cabins or on deck?" "On deck?" We all gazed in dismay at the deck. The rain was pouring down in torrents, as only tropical rain can pour down. The decks were literally swamped. "Our cabins?" The rain was beating in to such a degree that the port-holes had to be closed. The air of our respective bunks was something like that of the Black Hole of Calcutta. "Sorry, ladies and gentlemen," said the apologetic deck steward, "but the rules of the company don't allow any food to be served in the drawing-room." We appealed to the doctor. He assured us he was helpless in the matter. The company's rules must be kept by the ship's officers —even by the doctor against his better judgment. One of us had a bright inspiration. "Put our deck chairs in the companion way, and we can take our soup in the fresh air there." This was a colloquial way of putting it. You don't get much fresh air in the tropics at the beginning of "the rains." "Sorry again, sir, but the Captain's given strict orders that none of the passengers' chairs are put in the companion." The doctor corroborated this most vexatious of "ship's orders." The upshot of it was we sampled our cabins below, found them so stifling that we decided to run the risk and take our broth and pudding on deck. Each sat under an open umbrella on the sloppy deck, the rain pelting down on us through the thin canvas awning above. This was our first dinner on board. The typhoid patient had a bad "go" of malarial fever on at the time. I took his temperature just before dinner, and it was 103°. Everybody knows the danger of a chill in dysentery. The day I sailed my doctors told me a chill on board would probably prove fatal. The miscarriage case was obviously in great danger. We all wished we could take a snapshot of that *al fresco* invalid dinner for exhibition at the Royal College of Physicians. The typhoid patient kept to his bunk for the next two days with relapsing fever. Is it surprising?

### "These Terrible Meals!"

I was terribly ill for many days after this remarkable dinner on deck. I fear some readers may be tempted to doubt the statement, but no alteration of the rules took place for the rest of the voyage. Wet or fine we took our meals on deck, or in our stuffy bunks, with the option of the full dress saloon table. It poured almost incessantly till we got to Aden. The doctor and captain both maintained they were powerless to permit us to take our soup in the deck saloon, or to sit in the companion way. I shall never forget those terrible meals in the blinding rain, on deck, or in a stuffy cabin in the suffocating Red Sea, the porthole often shut, on account of the drenching downpour. The smell of the food, the interminable courses, and the inevitable dressing at the saloon table, make eating a most wearisome ordeal to the invalid, and it seems a simple matter to grant a comparatively cool, invalid corner on one of these big boats from the East carrying, as most of these do, at certain seasons of the year, a big freight of sickly Anglo-Indians and delicate women and children. In justice I must remark that the food was almost faultless. I never came across such admirable sea-going sick cookery as this steamer furnished. One had only to ask for special foods and they were served promptly and of admirable quality.

OTHER HARDSHIPS.

Our misfortunes did not end with discussing our ménus in the rain. There were other points of manifest discomfort for the invalid. My cabin was amidships fore of the saloon, my two invalid companions in distress were berthed close to me. Our cabins were good, but the lavatories and bathrooms in that section of the ship were locked and the keys held by the captain. It is hardly necessary to point out the great hardships resulting to invalids from such an arrangement. We had constantly, in dressing-room attire, to pass through the saloon full of passengers on our way to bathrooms and lavatories. We appealed to the doctor to explain the hardships of our position to the captain. The official answer was that the lavatories and bathrooms were always locked unless a certain number of passengers were berthed in that section. To people in our state of health that long walk to the other side of the ship at all hours of the day and night was a very serious consideration — apart from the unpleasant publicity involved.

SPECIAL PROVISION WANTED.

I will pass over a dozen other little conditions which made the position of board-ship invalid very hard to bear. I have simply set down the main cause of my complaint, that sick persons are not properly catered for on board ship. It is so universal nowadays for doctors to prescribe sea voyages both for really sick and for convalescent patients, that special provision should be made for such cases. Since my experience of returning invalided from the East, I feel heartily sympathetic towards sick people who go down to the sea in ships whose company's rules are of the vexatious type I describe. Be it remembered that this was a crack ship of a crack line, on a route where foreign competition is rapidly winning favour. I have been on many steamers in many parts of the world, but never before as an invalid, so I cannot say from personal experience how other lines compare with this in the matter of catering for the sick. But I heard constantly from fellow voyagers on board my boat that passengers sailing under a foreign flag were not hemmed in by so many rules and regulations as we were. In view of the many dangerously sick persons returning each year from our possessions in the East, it is of the utmost importance that proper provision should be made for their comfort and needs. In view also of the many invalids ordered "sea voyages for health," it is of the utmost moment that they should be able to command on board ship a few of the comforts essential to sick people. If sea-going invalids are to be treated as were the sick trio I write of, they had far better stay on dry land. But the sick man of the East has no option. He is peremptorily ordered out of the tropics. However ill-adapted a steamer may be to his requirements, however meagre the accommodation, however prison-like the regulations, he is bound to take "passage home."

A NURSE FOR EVERY STEAMER FROM THE EAST.

Hereafter I will point out some of the practical lines on which a system of sick nursing at sea might be established. I have seen so much pitiful suffering on steamers among young men returning sick from China, the Straits Settlements, and India owing to the lack of an official nurse on board, that I registered a vow on my last voyage from the East in a ship full of such invalids and semi-sick that I would set my pen to advocate the necessity for a certificated nurse on every steamer bound from the East to London.

*(To be continued.)*

### Day and Night in a Maternity Hospital.

BY A CORRESPONDENT.

THE gloomy old building, on which soot, smoke and fog had worked their will for many a long year, did not particularly impress us as we drove up and alighted. Within we were met by the porteress and ushered into a long barely-furnished room adorned by pictures of women and children, dingy with age. Here we were left in the care of a nurse. She ran (all the nurses we met seemed to have forgotten how to walk), and we followed, flight after flight, until at last, nearly breathless, we entered a large room divided into four by curtains. Two nurses slept in each of two divisions and four each in the other two. "Put on your uniforms as quickly as possible, supper is nearly ready, and you are not allowed to wear anything else," said our guide. The process completed, we surveyed ourselves half laughingly in the unwonted gear, and followed her once more. We prowled up and down the corridors, where the nurses gazed at us with suspicious eyes, none volunteering to speak until the supper bell rang, and we trooped down one corridor after another to be at last greeted by hash, bread, butter, and water for those who did not care to pay extra for beer or milk. After prayers we retired to bed. "You will probably be called up in the night, as you are fresh, so you need not take your clothes off," was the parting advice proferred by one of the nurses, with a grin; and "you will only have three minutes to dress in," volunteered another. The door bell rang once or twice in the night, and each time we jumped up, but no one came for us and we lay uncomfortably until morning.

THE LABOUR BELL.

Next day my turn came. At the sound of the labour bell, everything was dropped and away we rushed. Directly the baby was born it was placed in the incubator, the mother attended to, and then came the washing of as slippery a bit of humanity as it is possible to find. It wriggled and twisted, and the more it wriggled the hotter I grew. At last the dressing process commenced, and I, who had never seen a baby's dress, had to have all the different garments named and be shown how to arrange them. I was thankful when the ordeal was over. The chair on which I sat was an inch or two higher than the basin, and at each movement I unconsciously grabbbed the infant to prevent it rolling into the bath. Stores are given out directly after breakfast, and consist of eye and mouth rags, pads, tow, soap and powder if required. Each nurse takes her own stores, and the tow bag is in charge of the nurse on ward duty.

THE DAY'S ROUTINE.

On entering the ward the hands are dipped in mercury and not dried, and then the business of the morning proceeds. Temperatures are taken, mothers washed and douched, beds made, all the utensils disinfected and cleaned, bed-pans polished with brickdust and tow—alas, our poor hands!—and then comes the babies' turn. Four are washed at a time by their respective nurses at a low square table with sunk basins. The babies take advantage of being undressed to expand their lungs, and for a time Babel reigns. The nurse on ward duty brings in lunch, flowers, and plants, and after a general wash of baby clothes, etc., the nurses may probably have a few minutes' rest. After dinner and the daily visit of inspection, blinds are closed and the patients generally have a little nap. Tea about three o'clock for the mothers, and at five o'clock the morning's routine starts again. Supper and prayers wind up the day, and bed is thankfully hailed by the wearied nurse. Of course it is quite probable that she may have to get up to a case once or twice in the night, and if it is hers she will have to clear up, wash the baby, and consider herself very fortunate if all is finished in an hour and a half. Sometimes a nurse more good natured than the rest helps with the cleaning and disinfecting.

Night Work.

My friends pitied me when I went on night work. They were sure I should not like my companion nurse, and as I found out afterwards she was dreading my arrival just as much. We discovered our mistake, and in the end parted with mutual regret. After supper we made our way to the pokey little kitchen upstairs, where the meals were prepared for the night superintendent, ourselves and patients. It also acted as a sitting-room when we were not answering the door bell, receiving patients, and attending to the wants of the babies and their mothers. It is an eerie sort of feeling in the middle of the night, with doors and windows wide open, and an unearthly stillness brooding over all, to be suddenly roused to the commonplace by the clang of the front door bell. Both nurses rush down. If it is a case the patient is directed to the side door where one nurse receives her whilst the other goes upstairs and informs the night super. The "lady" is undressed in the receiving room—all patients are styled either "waiting lady" or simply "lady"—and reclothed in hospital attire, consisting of grey stockings, carpet slippers, night-dress, petticoats, and dressing-gown. Thence she is escorted to the labour room, where she is examined, an enema and bath administered. If the case is progressing, two midwives are summoned to make an examination, and later when the head is on the perineum, the nurses are roused, generally to find the baby on the bed. The regular work goes on all the time; meals are carried round; first babies are taken out of the incubators and left with their mothers to be fed, re-collected after a short interval and the mother's wants supplied. As the night advances the nurses feel less and less sleepy until at last when we meet our friends amongst the day nurses at half-past eight, we can hardly realise that bed is due in an hour or two.

### "Study what you most Affect."

It is very interesting to a nurse in reading Shakespeare to see how many allusions he makes to sickness and the arts of medicine and surgery. There is not one of his plays in which one does not come across such lines as

"What wound did ever heal but by degrees?" ("Othello"); or

"If they had swallowed poison 'twould appear
By external swelling." ("Antony and Cleopatra.')

He observed and noted symptoms with as keen an eye and as eloquent a pen as he watched and recorded every motive and act of his fellow men, and every beauty of landscape and charm of season. What nurse might not mentally echo his words a hundred times over at the too familiar spectacle of the garrulous patient whose chief joy is in recounting his ailments? "I am not very sick, since I can reason of it," he makes Imogen say; as we say now, "She cannot be very bad, or she would not talk about it so much." And Mrs. Quickly's description of Falstaff's death appeals to everyone who has seen the end of a fatal chronic illness as being exactly true to nature:—

"After I saw him fumble with the sheets and play with flowers and smile upon his fingers' ends, I knew there was but one way; for his nose was as sharp as a pen and 'a babbled of green fields . . . . so 'a bade me lay more clothes on his feet: I put my hand into the bed and felt them, and they were as cold as any stone; then I felt to his knees, and they were as cold as any stone, and so upward and upward, and all was as cold as any stone."—("Henry V.")

He makes another allusion in "King John" to the quiet muttering delirium of the dying:—

"His pure brain
. . . Doth by the idle comments that it makes
Foretell the ending of mortality."

When Cordelia dies, her father exclaims—

"Lend me a looking-glass;
If that her breath will mist or stain the stone,
Why, then she lives."

So Queen Katherine's attendants ("Henry VIII.") remark on her changed appearance as a sign of death—

"Do you note
How much her grace is altered on the sudden?
How long her face is drawn? how pale she looks
And of an earthy cold? Mark her eyes!
She is going, wench: pray, pray."

Besides being familiar with the sights and sounds of the sick-room, Shakespeare was acquainted with some, at any rate, of the materia medica of his age; even in the Sonnets comes a reference to a popular remedy or preventive against disease—

"While like a willing patient, I will drink
Potions of eisel (vinegar) 'gainst my strong infection."

And in Sonnet 118 we find—

"To prevent our maladies unseen
We sicken to shun sickness when we purge."

While in the next Sonnet he refers—surely from some personal experience—to "potions . . . distilled from limbecks foul as hell within," which reminds one of the celebrated witches' cauldron in "Macbeth," whose ingredients were hardly more startling and improbable than those of many a prescription to be found in contemporary writers.

"First aid" must have been as uncertainly administered in Shakespeare's days as it is now by the unskilled. When Henry VI. swoons at the trial of his uncle the Duke of Gloucester, the Queen exclaims, "Help, lords; the king is dead!" Whereupon the Duke of Somerset offers the advice, "Rear up his body, wring him by the nose," on the principle, perhaps, that it is better to do something than to do nothing. But when Othello falls into "his second fit," and Cassius recommends rubbing the patient's temples, the advice Iago gives is quite to the point.

"No, forbear;
The lethargy must have his quiet course . . .
Do you withdraw yourselves a little while,
He will recover straight."

Juliet's nurse, seeing her mistress lying apparently dead from the effects of the sleeping draught she has swallowed, cries out, as nine out of ten uneducated women of her class would cry out in these days, for brandy to revive her. Earlier in the play the nurse has complained greatly of aching bones, beating head, and pain in the back, for which her panacea was apparently a poultice, as she asks Juliet:

"Is this the poultice for my aching bones?"

The nurse grumbles like "one that's sick o' the gout," yet, says Posthumus in another play, she surely

"had rather
Groan so in perpetuity than be cured
By the sure physician, death."

There are a great many physicians to be found in the pages of Shakespeare: the "much-famed" physician who was father of Helena, the doctor who watched Lady Macbeth in her sleep-walking, Romeo's apothecary, and many others, but scarcely any sick-nurses are mentioned; for when Richard II. declares:

"I am too old to fawn upon a nurse,"

he evidently means an attendant upon children. There is, however, certainly one allusion to sick nurses in "Romeo and Juliet":

"How oft when men are at the point of death
Have they been merry which their *keepers* call
A lightning before death."

### "Elizabeth:" a Sketch from Life.

In the women's ward of a general hospital, lights are low and all settled for the night. A crimson glow falls across a bed in one corner from a little lighted window in the wall alongside of it; the window looks into a nurse's bedroom, for in the days this not very modern hospital was built, no regular night nurses were considered necessary. The day nurse slept in this room (which was, of course, provided with another window on the other side), and the little window was for means of communication when the patients wished to call her.

But now the window is fastened up and a red blind tacked across it. Its glow falls across the bed of "Elizabeth," a hopeless internal case, whose days are numbered. Bravely patient, unusually refined, her large dark eyes always pathetically thanking those who do their best to ease her pain, so that the nurses' voices take a tenderer tone when they come to her; she is so grateful, so afraid to give trouble, and so quick to feel their sympathy. Her very presence in the ward has good effect, grumblings cease and rough voices soften when she mildly says: "Well, well, 'twont be for long, you'll soon be well again," or reads to them in her plaintive voice some little thing out of the precious little book called "Daybreak," which she keeps under her pillow, and which contains a balm for many ills.

To her the window shines friendship and company, for though she never shows her preference openly, the nurse who occupies the room behind it is her most loved nurse of all, who, knowing that she watches the window, leaves the light as long as rules permit, and then before she goes to bed, taps softly through the blind "good night."

But this night Elizabeth is worn and weary with unusually severe pain, and when the light goes out, she lies long awake, thinking sadly of her home and children. How hard it is to leave them! How will they fare when motherless? And big sobs come, until at last she weeps in bitter hopelessness.

The nurse inside the window by some cause awakened, hears the bitter sobs, and listens for awhile, until she cannot bear it any longer; at length she rises and putting on her dressing-gown steals to the door. She hears the night nurses busy with a new arrival in the next ward, so she goes to Elizabeth and puts her arms around her. "Poor Elizabeth," she murmurs, and the sobbing woman clings to her, and tells the reason of her sorrow, and so she holds her closely until the sobs grow quieter, and cease.

"Dear nurse," the woman says, "how kind you are! I trust if ever you are sick or sad the dear God will send you a comforter, as He has sent you to me. I will be good, and now go back, I hear the sister coming, she will not be pleased to find you here."

A few days after and Elizabeth needed no more comfort. In the "Daybreak" book was written in faint and trembling pencil—"Nurse ——— from Elizabeth."

### The Nurses' Bookshelf.

Our Baby: For Mothers and Nurses. By Mrs. Langton Hewer. Seventh edition revised. (Bristol: John Wright and Co. 1901. Price 1s. 6d.)

A seventh edition does not want much review. This book has become a standard in many respects. It certainly is a most readable account of baby life, and gives a great deal of useful information in regard to the proper rearing of these delicate morsels of humanity. It seems highly charged with knowledge and common sense, and if a child is brought up with knowledge and common sense, what more can be wanted?

How to Nurse the Sick. The Home Series, No. I. Edited by Florence White. (London: Published at the Office of *Black and White*, 63 Fleet Street. Illustrated. Price 2d.)

The wisest of all men said many a year ago that "of the making of books there is no end." What would be his thoughts on the subject in the present day? In the matter of nursing handbooks alone the market is flooded, and they are almost all good—some more, some less. "How to Nurse the Sick" does not tell us much that we have not read hundreds of times before, but the hints are good, and if the amateur nurse is careful to attend to her instructions, and not to go beyond them, she will be found an efficient person. In the section on "fomentations" very good teaching is given; it is a pity that the illustration given does not quite agree with it, for it represents a proper fomentation wringer—an unnecessary item of which the text does not speak. Bed-making is well described, but the paragraphs on taking the pulse and respiration are not very useful to the amateur. Any doctor who trusted these duties to a home nurse would be indeed a confiding person. Such work is not for the untrained attendant. The same, we think, may be said of jacket poultices; they are not easy to make and apply even with skilled hands, and when manipulated by the inexperienced are likely to do more harm than good.

The Convalescent's Diet. By Lucy Helen Yates. (London: H. Virtue and Co., 26 Ivy Lane, Paternoster Row. 1901. Price 1s.)

This small book will prove useful both to professional and amateur. A really excellent nurse may be lamentably deficient in the gift that thinks out successfully a variety of dishes on a restricted dietary, and she will hail the unpretending little volume before us with rapture. First, there is a whole week's dinners arranged to follow a strictly milk diet, and if by Saturday the attendant is utterly unable to think of anything new, she can at least begin at Sunday again. Then there is a series of well-arranged diets for those suffering from various diseases, such as anæmia, diabetes, &c. . . . This is useful to all, but especially to the home nurse, who naturally does not understand much of strict dieting, and to whom the doctor (forgetful of this fact) omits to give directions, or having given his orders leave the items to the often sorely perplexed amateur nurse and cook. It is rather a pity that Miss Yates has not supplied a little information on the subject of feeding convalescents from typhoid fever; a question which presents some difficulties even to the skilled attendant. She gives some good hints as to preparing small appetising dishes of fish for a jaded appetite, and tells us how to dress up our old familiar friends in new garb and with fresh surroundings.

### Echoes from the Outside World.

### AN OPEN LETTER TO A HOSPITAL NURSE.

We all know how Queen Victoria employed her leisure moments in knitting for the benefit of the poorest of her subjects, and though one cannot help wondering whether our late Sovereign would have been glad that what she intended to be an article of use should be converted principally into an object for admiration, one can quite understand the sentiments of the Isle of Wight Board of Guardians. They have decided that the handsome quilt which was their last present from the Good Queen, and which was her own handiwork, shall no longer be allowed to serve only as a covering for a bed at the Parkhurst Workhouse Infirmary. It is to be placed in a suitable case and used as a screen, so that for many years to come the inmates may be able to look upon it and remember the kindly solicitude which was always shown them by the gracious lady who, as well as the Sovereign, was the personal friend of those fortunate enough to live in the vicinity of any of the Royal residences. This movement on the part of the Isle of Wight guardians will probably suggest to other institutions and to private individuals who own Royal gifts a similar employment of their treasures. Those who turn counterpanes into screens will probably plead that after all they only divert the usefulness into another channel. What was intended to add warmth is made to keep away cold, which comes, practical minds will contend, to much the same thing in the end.

---

It is only a truism that there are a large number of careless people in the world; but I think I had hardly realised how large that number was until I read this year's annual report of the business of the Post Office. I would not like to say that I had never posted an unaddressed letter, but I do not think I have committed such a sin of omission more than once. Yet no fewer than 345,690 packets were put into the letter-box in an anonymous condition during 1900, and the general public seems to be getting more, rather than less, careless. The number of articles so badly secured that they escaped from their wrappers shows an increase of 12 per cent. over those found last year. The coin alone which escaped from envelopes, etc., amounted to £902, and the total value of property contained in letters so insufficiently addressed as to be undeliverable, and consequently sent back to the Returned Letter Office, was £681,335. Also, it is a fact worth remarking, that the amount deposited in the Savings Bank continues to decrease year by year. In 1896 the decrease was £10,000, in 1900 it was only £5,000. There is, however, ground for hope that this is not altogether want of thrift, because the amount of Government Stock held by depositors has increased during the past year by £2,000, so evidently some of the saving ones preferred one kind of investment to another.

---

Weymouth was unusually gay on Saturday when the annual regatta under the flag of the Royal Dorset Yacht Club was held. It was a lovely hot day, and those who looked on thoroughly enjoyed the lazy *dolce far niente*, but the racers were sad because the wind, which promised at the first to be most satisfactory, fell off in a lamentable fashion, and it was impossible for the vessels to go the whole length of the course. *Magdalen*, owned by the Baron de Forest, was successful in one of the races, although she had unfortunately crossed the line just too soon and so had to go back and cross it again. This made her victory the more popular, and the fact of her having resolved to stay at Weymouth and race the new yacht *Quand Même*, rather than repair to Torquay to join in the regatta there, won her further encomiums. *Quand Même* has been built by the Duc Decazes specially to sail for the Coupe de France, and she arrived at Weymouth early so as to be ready for the Wednesday trial matches. Three are to be sailed unless either vessel wins the first two, which, of course, would be decisive. The weather was so stormy and boisterous at Torquay on Monday that we were particularly jubilant over the better fortune which had befallen us on the occasion of the Weymouth festival.

---

Amongst other excursions which are easily made from Weymouth, I must tell you a little about Lulworth. It is a sweet place, one of the prettiest in Dorsetshire, which, considering the numerous beautiful spots it can boast, is saying a good deal. There are frequent steamers, but as some of our party were not quite certain of their sea-going powers, we went by the South-Western Railway to Wool Station, and then an omnibus drove us five miles to the Cove. It is a pleasant drive over hilly country, but one is scarcely prepared for the beauty of the little place itself when one gets there. The sea is a most glorious blue, the rocks about are very fine, there are plenty of sights to be seen in the neighbourhood, and all around is peaceful and quiet, far removed from such delights as bands and piers, and Pierrot troupes, and therefore only suitable for those whose idea of holiday enjoyment is rest and retirement. Apartments are to be had, it is true, but they are so few in number, and so many people come year after year, that it is only by arrangement made long beforehand that rooms can be secured. The Bishop of Salisbury usually spends some weeks there. The Cove itself is a large round sort of basin which has been hollowed out of the cliffs by the action of the waves. There is an entrance from the sea at one side, and, though we did not bathe, arrangements seem excellent. I hate bathing away from home as it were, and going about all day afterwards with untidy hair and a generally uncared for appearance, besides the burden of a damp bathing dress. There are tents for ladies on the far side of the pool, and several sandy bathing beaches. Oswald Bay is an especial favourite with those who have their own portable tents. There are some wonderful caves to be seen, notably the Cathedral Cavern, and no one should even pay a passing visit to Lulworth without seeing Durdle Door. It is a large natural arch about 40 feet high, which is as neatly and evenly made as if a band of skilled workmen had done the work. The golf links in the neighbourhood are of course an additional attraction to visitors who play the game.

---

At the present time, when so many of us repair to the country or the seaside to apartments which we have engaged by letter and have had no opportunity of seeing for ourselves, the recent decision of a London magistrate on the subject is well worth noting. Two young Belgian ladies came on a visit to this country. A lady friend engaged apartments for them in a house in Sydney Street, Chelsea, which, as far as she could judge, appeared to be quite desirable. The travellers came by the night train, and upon their arrival, tired and weary in a foreign land, they drove at once to the refuge which had been prepared for them, only to be met by a landlord who was half tipsy and wholly abusive. So unsatisfactory did they find their quarters that they left before the evening, but the landlord demanded a week's rent before he would allow their boxes to be taken away. Fortunately, the two Belgian ladies, instead of weakly parting with two guineas in order to get out of such an uncomfortable situation, as I fear most women would have done, applied to the Court at Westminster for assistance. A constable was at once sent to recover the luggage, the magistrate stating that it is a fact which should be known that luggage cannot be detained unless a week's rent be already due. I think this information may be useful to many of us at some time or another. I know of several cases where friends have engaged apartments beforehand, found them most abominable, and yet put up with them, or paid the full amount which would be due at the end of their visit rather than risk having to leave without their luggage. With the feeling that the law is on their side it is possible that at least some of them may now take their courage in both hands and boldly make a move when things are beyond bearing, buoyed up by the knowledge that if any attempt is made to deprive them of their boxes the police-constable will come to the rescue.

### Everybody's Opinion.

[Correspondence on all subjects is invited, but we cannot in any way be responsible for the opinions expressed by our correspondents. No communication can be entertained if the name and address of the correspondent is not given, as a guarantee of good faith but not necessarily for publication. or unless one side of the paper only is written on.]

### VENTILATION AND FRESH AIR.

"L. H." writes: I should like to know what the editor of the NURSING MIRROR thinks, and what nurses' experiences are. But I think that builders are behind the times in matters of ventilation, hygiene, etc. Some time ago I went to see a nurses' cottage attached to some almshouses; the cottage was having extensive alterations, and a room (ward) attached to the kitchen had been recently built, yet, there was only a window in the room, and no other door, except through the kitchen. Now all the bed-pans, etc., would have had to come through the kitchen. If the patient died, the corpse would have to be brought through, and there was not a ventilator in the place, and no glass door to see how the patient was getting on. I mentioned these facts at headquarters, and things may have been remedied. But where nursing homes of any kind are to be erected, I think the builder should understand that it is not an ordinary house he is building. I believe that much illness could be averted by having a better, at least a fuller, system of ventilation in our big towns. Public parks and open spaces are a blessing, but it is the few and not the many who go in for fresh air in their houses. Perhaps, as the future of nursing turns to an open-air system largely, the public will realise that fresh air and better ventilation will help to prevent illness, as well as helping to cure it.

### PRIVATE NURSES' FEES.

"NURSE D." writes: I feel as "A Sympathiser" does that a nurse ought to have the full benefit of the fee paid for her services. If a nurse is conscientious, nursing taxes the strength perhaps more than any other work. If a typist can earn £200 yearly for five or six hours daily, surely a nurse is underpaid with a salary of £30 for 16 or 17 hours a day, sometimes taking night watching as well. I think the reason why so many nurses join institutions instead of taking cases on their own responsibility is the fear lest they should be without cases for a length of time which would make their salary even less than £25 or £30. I think a nurses' club a very good idea, as, if seven or eight certificated nurses lived together in a house or home, doctors would look upon it as a nursing institution, and would of course send there for nurses.

[Our correspondent has been misinformed about the earnings of typists, which average about £1 a week, while the hours are often as long as those of nurses.—ED. HOSPITAL.]

"NURSES' HOME" writes: As being connected with one of the much maligned nurses' homes, will you allow me to say a few words on the other side of the question? In the first place our nurses cost us at least £40 for training, salaries, and uniform before they come into the home at all, naturally we want some return for the expenditure. Their salaries range from £20 to £35, exclusive of uniform and occasional bonuses. They have from three weeks' to a month's holiday every year, during which time they of course earn nothing, and at least two days—however busy we may be—between each case, be the latter ever so short. If they are ill, they have free nursing and every care, have an extra holiday in which to recruit, and frequently seaside lodgings are provided. They are also given 5 per cent. on their savings. When you deduct all this, with uniform and board, from the £80 mentioned, but which sum could only be earned if the nurses were employed every day of the year, the profit to the home is not more than sufficient for rent and management; and if they are so badly treated why do most of them stay ten to twenty years, while those who do leave come back to ask for work?

### MONTHLY NURSES.

"MOTHER" writes: For many months my mind has been greatly exercised upon the subject of monthly nurses in their relation to the infant. My experience is that the ordinary trained (three months) obstetric nurse, until she gains her knowledge by years of experience, is very ignorant of the manifold needs of the twentieth century—and, alas! invariably bottle-fed—infant. Being in the position to view the question from the standpoint, first of nurse and now of mother, I feel at liberty to comment upon it. The term of training is short, too short to enable the woman of mediocre intelligence and education to grasp all she requires to know to enable her to satisfy her examiners before leaving her hospital. So naturally the mother, and rightly too, occupies all her time and thought while there, and her duties in this respect she fulfils carefully and conscientiously, and undoubtedly she would ungrudgingly bestow the same care and attention upon what she considers a very secondary part of her work, had its duties been drilled into her as unceasingly and unsparingly as were the others. Writing from my own experience (and I am sure other nurses have found it theirs) I am persuaded that it is not until we are fully launched in our private work, with its terrible weight of responsibility and anxiety, that we become alive to the important part the baby now plays. If baby is well and strong and nature has kindly supplied an ample supply of food our duties are comparatively easy; but what of poor baby when it must be artificially fed and nurse's knowledge ends with "one part of milk and two of water, etc.?" Happy the nurse whose charge is obliging enough to thrive upon this! Alas! many don't! The bright intelligent reading woman who has kept her eyes and ears open, ever gleaning golden information from every available source, can generally cope with these difficulties, and with great care and patience overcome them; but then there are the vast majority of "rule-of-thumb" nurses. In respect to these I say, give them a few more rules. Keep baby before them a little longer; give them a few opportunities of cleaning bottles and preparing food; let the sisters and staff nurses in our hospitals be as unremitting in instilling into their pupils the needs inward and outward of their tiny charges as they are in enforcing careful attention to the mother. Then, I think, the general public will benefit, much suffering will be spared, many lives will be saved, and the mother as she lies watching with eagle eye every movement of her nurse, will rest in peace and confidence that her precious darling is having bestowed upon it all that womanly love, knowledge, and science can suggest. The question, however, arises, Where is this extra knowledge to be gained? Every nurse of some years' experience who comes into contact with the newly-fledged nurse, or who remembers her own early struggles, will agree with me that the nurse has little time to think of the baby during her training. In our ordinary Lying-in Hospitals the baby goes out in 10 days or so, and during that time has been fed by its mother. How would it do to supplement the three months with one or two in a Children's Hospital, where the infants in these institutions would be entirely in their charge? Motherhood has opened my eyes to the necessity of better training, and caused me to think a very great deal about it. In fact, none can deny that there is a need, but the remedy is not so clear.

### BRITISH NURSES ABROAD.

"A NURSE" writes: I wonder why more British private nurses who have saved a little do not pack up and go to some places abroad where nurses are needed so badly, instead of sitting and waiting for cases to "turn up" at home. Certainly it requires no small amount of pluck to go to a foreign land where one has no friends and cannot speak the language; but from my own experience I am certain that a well-trained capable woman is appreciated everywhere; and especially in parts where people often die for the want of a little experience and care. I have just returned from Sao Paulo, in Brazil, where I went with the intention of having a long much-needed rest with my hospital chum who has married and settled out there; but a fortnight after my arrival I heard that an Englishman was seriously ill with hæmorrhage in a hotel with only native servants to see after him. I gladly went off at once, and never shall I forget his great appreciation, for he had been two days on his back without a wash or his bed touched because "he was frightened to move." I was with him six weeks, and during that time picked up quite a number of Portuguese words, as my patient was not allowed to speak, so I was obliged to ask for what I wanted in the hotel. During that time I was fetched for a confinement case, which I had to manage entirely alone with only a little Italian servant in the house who could not speak a word of English, and then went backwards and forwards in the day and managed both patients, not forgetting the baby, simply because there was no other nurse to help me. Portuguese is the native language spoken and it is easy to pick up; the English children speak it before their own tongue. French and German is also most useful; in fact one seemed to hear a little of everything spoken. There is one English midwife who is able to keep herself and send home enough to support her five children as the fees are very good. Midwifery cases are paid for at the rate of 300 to 350 milreis for ten days, and a milreis was worth over a shilling when I was there, so in less than two months I was able to pay all my travelling expenses first-class return, and also my outfit, but, of course, as I was stopping with my friend I had no board or lodging to pay. Sao Paulo is two days' journey by land from Rio, but I landed at Santos, and then went up in the cable train; it is one of the most beautiful railways in the world, thousands of feet high, and grand scenery, some parts being only inhabited by monkeys and parrots. The climate is healthy compared with other places near; some enteric, but only a few cases of yellow fever which go into the fever hospital, which is managed by Brazilian doctors and an English staff of nurses; there is also an English general hospital, but they have not any nurses they can spare to send out to cases. The Brazilian people are said not to appreciate a trained nurse, but I nursed one Brazilian lady, and, although our conversation was not fluent, she was most grateful and pleasant, and said when I left "she would have longings for me." When a Brazilian gets ill all the relations come in by the dozen; they come first thing in the morning, take off their hats, without being asked, and stop on nearly the whole day; but on that one occasion they seemed a little awed by the English nurse, and I really had no trouble to keep my patient quiet. At some of the Brazilian ports where we stopped I heard sad stories of the need of nurses, especially midwives. Surely some of our home nurses could be spared and would not mind venturing if they knew that they were needed badly among the English inhabitants.

[It must be noted that our correspondent went out to stay with a friend, and we can but repeat the advice we have often given before that unless they have either means or friends it is unwise for nurses to go to foreign countries on speculation. Make inquiries at the Emigrants' Information Office, 31 Broadway, Westminster, S.W.—ED. "THE HOSPITAL" NURSING MIRROR.]

### Appointments.

ALMONDSBURY MEMORIAL HOSPITAL, BRISTOL.—Miss Annie Tanner has been appointed matron. She was trained at the Bradford Royal Infirmary, where she was gold medallist for 1897-8. She was afterwards charge-nurse at the Rochdale Infirmary, and for the past two years sister of the female medical and gynæcological wards at the General Hospital, Wolverhampton.

BRITISH LYING-IN HOSPITAL.—Miss Alice Sanderson has been appointed assistant matron. She was trained at St. Thomas' Hospital, and afterwards held the post of sister at the Hospital for Women and Children, Waterloo Bridge Road. She holds the certificate for midwifery of the British Lying-in Hospital and the L.O.S. diploma.

CROYDON GENERAL HOSPITAL.—Miss Nora Smythe has been appointed sister to the children's ward at this hospital. She was trained at the Great Northern Central Hospital and qualified at Queen Charlotte's Hospital for the L.O.S.

DENBIGH INFIRMARY.—Miss Picton Davies has been appointed lady superintendent. She was trained at the Royal Devon and Exeter Hospital, and has since been attached to the Surgical and Medical Nursing Hall and Midwifery Training School, Walthamstow.

DEVON COUNTY COUNCIL.—Miss Ada Fricker has been appointed staff instructress in nursing. She entered Guy's Hospital in 1891, and has worked in connection with it ever since. For some time past she has been engaged in training pupils for the L.O.S. in the district covered by the Guy's Lying-in Charity.

SOUTH-EASTERN HOSPITAL, LONDON.—Miss Agnes Clark has been appointed charge nurse. She was trained at the Stanley Hospital, Liverpool, where she subsequently held the posts of charge nurse, night sister, out-patients' sister, ward sister, and theatre sister. She has since been engaged in private nursing in Scotland.

UNION HOSPITAL, GLOUCESTER.—Miss M. H. Jones has been appointed charge nurse. She was trained at Lambeth Infirmary, and worked there for four years. She has since been engaged in private nursing.

UNION INFIRMARY, MIDDLESBROUGH.—Miss Margaret Birkitt has been appointed charge nurse. She was trained for three years in the same institution.

WOLVERHAMPTON AND STAFFORDSHIRE GENERAL HOSPITAL.—Miss Alice A. Warriner has been appointed sister. She was trained at Grimsby and District Hospital and the London Temperance Hospital, and has since been charge nurse at Croydon Borough Hospital, sister at the General Hospital Devon, holiday nurse at Woolwich Cottage Hospital, and nurse at the Royal Hospital for Women and Children at Bristol.

### Death in Our Ranks.

WE regret to learn of the death of Miss Elizabeth Whitehead, superintendent nurse at Chelmsford Workhouse Infirmary. Miss Whitehead, who was 58, was a native of Burnley, and received her training at Netley Hospital. She had been superintendent nurse at Chelmsford Workhouse Infirmary for 16 years, having been appointed on May 19th, 1885. She gave the Guardians every satisfaction, and was esteemed both by the officials and inmates. The cause of death was an abscess on the lung, following upon pleurisy.

### TRAVEL NOTES AND QUERIES.

OSTEND OR SCHEVENINGEN (Poppy).—I think it will be too late for either to be very enjoyable. Ostend is much the gayer place and also much the more expensive, but I fancy Scheveningen would be better on account of its nearness to the Hague. A town is far preferable at that season. Why not stay at the Hague, where there is so much of interest, and go as often as you feel inclined to sniff the sea-breezes.

CIRCULAR TOUR FROM OSTEND (Wyeside).—By no means too late in the year. I wish you had told me what you could spend; without that knowledge my help can only be vague. I do not advise your trying to combine Holland and Belgium and starting from Ostend, the distance of Ostend from Holland makes it expensive, why not do either Belgium or Holland, but not both. As you read in my article, you can go from London to Rotterdam and back for 17s., and you could see everything of importance that I mentioned from Rotterdam itself. If you go to Belgium you can start from Ostend, but there are no combined tours for both countries starting from that port. Write me again directly you see this, and I shall have time to answer you. I am away from town, which causes delay. Tell me how you decide, and how much money you can spend, and I will advise you as to route and hotels.

OSTEND ON LOW TERMS (Leeds).—It is absolutely impossible to get accommodation in Ostend for £1 1s. per week. It is one of the most expensive places on the Continent. The very lowest terms I know of in Ostend at a modest hotel are £2 5s. per week. Lodgings, too, are very dear. Why do you fix upon so expensive a place? I must tell you, however, that I know of no place where you could live for such a sum as you name. Could we discover such a halcyon locality all the world would flock there. Tell me fully your plans, and the means at your disposal, and I will try to advise you.

PENSIONS IN PARIS AND BRUSSELS (Sister Vera).—I cannot encourage you much in your idea of getting cases in Paris. There are well-known nursing homes there, and I greatly doubt a stranger having any success. I must say the same of Brussels. As to pensions in Paris, I am away for my holiday, and therefore, not having papers at hand, I am obliged to trust to my memory. Try Miss Willoughby, 4 Rue des Pyramids, Paris. There is another one at 157 Faubourg St. Honoré, I cannot remember the lady's name. Address your letter Madame La Proprietaire. Both these are very reasonable. In Brussels try Hôtel du Rhin, 14 Rue de Brabant; ask for room on third floor, pension terms 5 or 6 francs per day, or Hôtel de Bavière. These are German in character, but most. respectable. Pensions are rather dearer, but if you like to try one Mme. Schürmann, 14 Rue d'Orleans, will take you from 7 francs per day.

### For Reading to the Sick.

THE BOND OF BROTHERHOOD.

I BEG of you calm souls—whose wondering pity
Looks at paths you never trod;
I beg of you who suffer—for all sorrow
Must be very near to God—
And the need is even greater than you see—
*Pray for me!*

I beg of you who stand before the Altar,
Whose anointed hands upraise
All the Sin and all the Sorrow of the Ages,
All the Love and all the Praise,
And the Glory which was always, and shall be—
*Pray for me!*
*A. Procter.*

Love for one—from which there doth not spring
Wide Love for all, is but a worthless thing. . .
But our pure Love doth ever elevate
Into a holy bond of brotherhood
All earthly things, making them pure and good!
*Lowell.*

But all Crowds are made
Of Individuals; and their grief, and pain,
And thirst, and hunger—all are of the One,
Not of the Many. And the power that helps,
Enters the Individual, and extends
Thence in a thousand gentle influences
To other hearts.
*MacDonald.*

Jesus Christ's revelation of the Fatherhood of God contains the disclosure of man's true relation to his fellows. In manifesting God as the universal Father, He taught men their common relation as brethren. The doctrine of Christ is, "God is your Father, therefore, as children of your Father, ye are brethren." Jesus Christ introduced a new spirit amongst mankind, the spirit of humanity. The life of sonship is the life of perfect relationship to God as a Father. As a consequence of this truth, it is also the life of fraternal fellowship with mankind, as children of a common Father. "Have we not all one Father? Hath not one God created us?" "If God is my Father, all His children are my brethren." Our Blessed Lord, by explicitly teaching us to address God as *our* Father, has implicitly bidden us to regard all men as brethren.—*Vernon Staley.*

We are apt to feel as if nothing we could do on earth bears a relation to what the good are doing in a higher world; but it is not so. Heaven and earth are not so far apart. Every disinterested act, every sacrifice to duty, every exertion for the good of "one of the least of Christ's brethren," every new insight into God's works, every new impulse given to the love of truth and goodness, associates us with the departed, brings us nearer to them, and is as truly heavenly as if we were acting, not on earth, but in heaven. The spiritual tie between us and the departed is not felt as it should be. Our union with them daily grows stronger, if we daily make progress in what they are growing in.—*Wm. E. Channing.*

### Notes and Queries.

**The Editor is always willing to answer in this column, without any fee, all reasonable questions, as soon as possible.**
**But the following rules must be carefully observed:—**
**1. Every communication must be accompanied by the name and address of the writer.**
**2. The question must always bear upon nursing, directly or indirectly.**
**If an answer is required by letter a fee of half-a-crown must be enclosed with the note containing the inquiry.**

*Probationer.*

(210) I should be pleased to know the names and addresses of general hospitals in London where I could apply for situation as probationer. My age is 31.—*E. H.*

See "The Nursing Profession: How and Where to Train."

Would you kindly tell me if a certificate from a small provincial hospital is of much value?—*G. M. H.*

Not unless the hospital is a school for nurses recognised by the Local Government Board. See reply to *E. H.*

*Incurable.*

(211) Can you tell me of any home which would take charge of a woman suffering from lupus on hand and face until her death? The disease is of too long standing to admit of any hope of cure.—*M. B.*

Much depends on the nature of the case. Your best plan will be to submit full details to the various hospitals and homes for incurables, for list of which see "Burdett's Hospitals and Charities," p. 966.

*Addresses.*

(212) Will you kindly tell me where I can hear of a cottage nurse, Holt-Ockley system?—*H. P.*

From the Secretary, the Affiliated Benefit Nursing Associations, Central Office, 12 Buckingham Palace Road, S.W.

Would you kindly tell me of an orphanage or home where I could get a young girl to assist in light housework and in the care of an infant in return for a good home and small salary?—*E. L.*

You will find lists of such institutions in the *Englishwoman's Year Book.*

Will you kindly let me know if Mr. Andrew Carnegie, the millionaire, has a residence in town, and tell me if you know his present address?—*L. C.*

We cannot give private addresses.

How can I get information about the (1) United Sisters' Friendly Society and (2) the National Deposit Friendly Society?—*E. S.*

Apply to the Secretary (1) 7A Lower Belgrave Street, S.W.; and (2) 11 Red Lion Square, Holborn, W.C.

I am anxious to join the Guild either of (1) St. Veronica or (2) St. Barnabas, will you kindly tell me where to apply for particulars?—*Nurse Winnie.*

From the Secretary (1) the Sanatorium, Clifton College; and (2) the Nurses' Hostel, Francis Street, S.W.

*Hip Disease.*

(213) Will you kindly tell me where a girl of fifteen, badly deformed with hip disease of ten years standing, without any education, poor and neglected, can be received and trained for £1 5s. a month?—*G. B.*

Perhaps the girl is suitable for the Cripples' Home and Industrial School, 17A Marylebone Road, N.W.

*Self Starvation.*

(214) I would like to know what is the best dietary for an aged patient who takes her food one day and rejects it the next?—*A. P.*

This depends upon the disease from which the patient is suffering. Consult a doctor.

*Tiptoe Exercise.*

(215) Will you please inform me about the "Tiptoe Exercise" as mentioned in the NURSING MIRROR of July 20, 1901.—*Sister G.*

It consists simply in the raising yourself on tiptoe several times in succession whenever you have a few minutes to spare. This action brings into play the muscles at fault, and thus strengthens them. But it must be persevered in for some time.

*Deaf Cure.*

(216) Kindly tell me if you can give me any information about ———'s electrical machine for the deaf. I enclose extract from paper describing it.—*C. E. M.*

If you are deaf you should consult a doctor.

*Coaching Lectures on Obstetrics.*

(217) Will you kindly tell me where I could obtain six coaching lectures on obstetrics from a qualified doctor in Wimbledon, Raynes Park, or anywhere between Sutton and Waterloo; if not, where in London, at moderate charge?—*Mary.*

We cannot name individual teachers. You might apply to the medical men in your own locality, or advertise in the local papers.

*Book on Dispensing.*

(211) Can you kindly tell me of an elementary book about drugs, their names, and how to dispense them?—*Nurse Anne.*

"Notes on Pharmacy and Dispensing for Nurses," by C. J. S. Thompson, would probably suit you; it is published by the Scientific Press at 1s.

**Standard Books of Reference.**

**"The Nursing Profession: How and Where to Train." 2s. net; post free 2s. 4d.**
**"Burdett's Official Nursing Directory." 3s. net.; post free, 3s. 4d.**
**"Burdett's Hospitals and Charities." 5s.**
**"The Nurses' Dictionary of Medical Terms." 2s.**
**"Burdett's Series of Nursing Text-Books." 1s. each.**
**"A Handbook for Nurses." (Illustrated.) 5s.**
**"Nursing: Its Theory and Practice." New Edition. 3s. 6d.**
**"Helps in Sickness and to Health." Fifteenth Thousand. 5s.**
**"The Physiological Feeding of Infants." 1s.**
**"The Physiological Nursery Chart." 1s.; post free, 1s. 3d.**
**"Hospital Expenditure: The Commissariat." 2s. 6d.**
**All these are published by THE SCIENTIFIC PRESS, Ltd., and may be obtained through any bookseller or direct from the publishers, 28 and 29 Southampton Street, London, W.C.**

### Travel Notes.

BY OUR TRAVELLING CORRESPONDENT.

### LXXX.—IN THE TAUNUS COUNTRY.

(*Continued from page* 272.)

EACH patient has his or her number on an ivory ticket affixed to the glass, but it is quite surprising how quickly the Priestesses of the Weinbrunnen and Stahlbrunnen recognise the various visitors and swoop unerringly upon your glass, without the necessity of your struggling to call out the number in probably very halting German. Some—generally those with a digestive trouble—are obliged to have the water warmed. I must say this is unpleasant, and it is more like stale soda water than before. The warming is most cleverly done by plunging the glass for about ten seconds into a metal box charged with steam.

The Priestesses have perforated trays that hold four glasses attached to a long handle, and these are plunged into the springs, bringing up the water cold and sparkling. The first sip, whilst the effervescence lasts, is pleasant, but one is particularly warned against a pernicious tendency to drink the whole at a draught. All must be done decently and in order, and we march up and down the woods in fine weather or the covered way in wet, solemnly sucking at our tubes?

There is no charge for the water at Schwalbach, but on leaving, it is customary to give a tip of 5 or 10 marks, according to your ability, to the priestesses. Admission to the drinking springs and bath-houses is covered by the cur tax (15 marks), which also allows of attendance at the concerts and dances and the use of the reading-room.

### THE BATH-HOUSES.

Of these there are several; the most convenient are those by the Weinbrunnen. The cost of each bath if you have a room with a south aspect is 2 marks, but if you are not particular about this, rooms with a north aspect are 20 pfennigs less.

Each room has a beautifully polished bath, and your attendant regulates the heat by a huge thermometer. I might rather say regulates the cold, for the baths are decidedly chilly, and as the cure advances you get it colder and colder.

Unless your circulation and state of health is very bad you stay in it quite motionless and with your head only emerging for 20 minutes. As you step in you shudder and feel that resting there five minutes will be insupportable; but no sooner are you completely covered by the water than bubbles form all over the surface of your body and a gentle warmth and feeling of exhilaration wraps you in a pleasant embrace. The grand thing is not to disturb the bubbles by the slightest movement. The attendant brings hot sheets and towels and entombs them in a hot metal box to be ready for your use; but if you prefer you can ring for him or her and be rubbed down vigorously. This is very desirable if your circulation is feeble.

At first it seemed a little startling that the attendants were impartially men and women; but I conclude that in the event of a bather ringing, they had a system of answering the bells with discretion.

I had a most venerable old gentleman as my slave; he had the appearance of a Baptist minister; it seemed quite out of place that he should be employed in such menial offices.

### IN AND OUT OF THE SEASON.

To those going for a cure I should strongly recommend the month of June. By going before the pressure of the season commences you have better and cheaper accommodation in hotels and lodgings, more attention from the doctors, and you can choose your own hour for your baths This last fact adds greatly to one's comfort. Later on crowds besiege the *guichet* at the bath house, imploringly seeking convenient hours and finding none, and the attendants overworked and overtired are not so agreeable.

Then, again, at the springs the throng is so great that one has to wait a very long time, and when the happy moment comes for coffee in the town, it is not infrequently impossible to find a table, especially if the weather is bad.

The walks are truly beautiful, and all over the Taunus district there is a system employed of marking the roads by different coloured bands on the tree trunks, most useful to pedestrians.

I longed to explore those lovely forest tracks more effectually, but during the progress of the cure one feels languid and disinclined for exertion—the reading-room (perhaps 100 yards from your hotel) appears to recede further and further from your view, and a descent into the village street to purchase ink or *Esprit du Bois* assumes the proportion of an Alpine expedition.

It is after the first ten days that one feels this washed-out condition the most, and one regards the row of Bath-chairs drawn up by the side of the Catholic church as articles of possible, and even probable, use.

But all this passes, and after three months a feeling of freshness and exhilaration sets in, though I think one does not realise the full benefit one has received until the end of six months.

There are some curious old houses in the town, when one has summoned up energy for the gigantic expedition involved in going to look for them, and a short drive takes one to Adolphseck, a very picturesque village with a ruined castle perched on a commanding height above.

### SCHLANGENBAD.

Five miles from Schwalbach is Schlangenbad, which has springs in every way different, and it is no uncommon thing for those who have been doing the cure at Schwalbach to go on to Schlangenbad to get rid of some external effects caused by the waters of the former place. In some cases the effect of the iron is so strong on the skin as to stain it a yellow colour, and though of course this effect very quickly subsides, devotees of fashion do not like to appear in society in so unbecoming a tint. It is by no means always the case—probably a particular kind of skin is needed to absorb the iron to the point of colouring it. In this dilemma nature has provided a remedy. The waters of Schlangenbad taken for ten days or a fortnight will remove all this appearance, and the skin becomes its natural colour and delightfully smooth and satiny.

It is a great remedy for some forms of skin diseases, especially those affecting the face, and also for debility and convulsive diseases. The Bath-house is more than 200 years old, and was built by the Landgrave Carl of Hesse Cassel, but there are additions of later date.

Not suffering unbearably from the yellow staining I did not stay at Schlangenbad, but only visited it two or three times when my energy was equal to a short drive.

At Schwalbach the mud baths are a great feature for those suffering from nerves, and the sufferers declare them to be delightful. It is a quaint process—you must keep your hands outside, or naturally the nails become receptacles for landed property, and on emerging you are cleaned down by the use of a species of vast watering-pot. All who have tried them declare the soothing effect to be quite magical; life seems suddenly to have become all *couleur de rose.*

